# Extracts

**Published:** 1882-08

**Authors:** 


### Extracts.

Treatment of Cicatricial Contractions.—Cicatricial contractions are the result of burns, of sloughs, of ulcerations, and of wounds, with loss of substance. They are caused by the exuberant growth of granulations, and subsequent contraction and induration during the healing process.

The following are the principal malformations resulting from the contraction of cicatrices: First. The margins of the orifices of mucous canals are everted, or drawn asunder, so that they cannot be closed. Examples of this kind are the wide separation of the eyelids, or of the lips, and the dilatation of the nostrils. Second. The orifices of mucous canals are contracted or closed; the eyelids, the mouth, the nostrils, the auditory canals, the anus, the urethra, the vagina, the œsophagus, and so forth, may be contracted in this manner. Third. The features may be disfigured by being drawn into abnormal positions. These distortions may affect the nose and mouth, the ears, the chin, and so forth, by being drawn out of their proper line. Fourth. Apposed cutaneous surfaces may become adherent. This kind of deformity is exemplified by the webbing of fingers and toes, by the adhesion of the arm to the thorax, or of the thighs to each other. Fifth. Fixation of one or more of the extremities in a position of exaggerated extension or of flexion, abduction or of adduction. Sixth. Permanent ridges on the surface, often more or less painful, and predisposing to cheloid, or other degenerations.

The next subject is the prophylactic treatment. When a surgeon is called to the treatment of a case in which there has been an extensive loss of substance, or destruction of tissue from any of the causes which have been indicated, he may employ either or all of the following means to guard against the occurrence of deformity. First, and this is a very important matter, the growth of exuberant granulations may be kept down by pressure and by the application of escharotics as often as may be necessary.

Second. The parts must be supported in such a position as to guard most effectually against the threatened deformity. Third. Incisions may be made from time to time in such parts as to favor the healing process, by contraction in the least injurious direction. These incisions may assume the form of **V** incisions, of ◊ incisions, of stellate incisions, or of circumvallation—that is, circular or oval incisions all around the affected part, so as to overcome for the time being the tendency to contraction. Fourth. Skin grafting may often be resorted to, with advantage, to form new centres for the healing process, and thus to diminish the amount of cicatricial contraction. Fifth. During the reparative process passive motion should be frequently employed to a very considerable extent, in order to stretch the newly-formed tissues, and to render them softer and more pliable.

When cicatricial contractions have already occurred, and have produced their characteristic deformities, appropriate treatment is to be employed, according to the character of the malformation to be overcome.

In the first class of deformities—namely, that in which the margins of the orifices of the mucous canals are everted or drawn asunder, the cicatricial bands are to be freely divided, so that the orifices can be completely closed, and a flap of sound integument dissected from the neighboring part and inserted between the edges of the wound, so as to fill up the chasm produced by the closure of the orifices. The flap thus inserted should be of ample dimensions, so as to allow for the contraction which will subsequently take place. The flap which is transplanted should always be somewhat larger than the space it is designed to fill; the pedicle should be of sufficient breadth to maintain the vitality of the flap, and a sufficient thickness of sub-cutaneous tissue should be raised with the integument, to secure a good vascular supply. The flap may be transplanted by the Indian method, that is, by twisting the pedicle on its apex, or by the French method, gliding the flap along without a twist. When the latter method is adopted, it is often advantageous to curve the pedicle, as by this means the tension is greatly diminished, and the flap is more readily brought into its place.

In the second class of deformities, in which the orifices of the mucous canals are closed, or greatly contracted, simple or multiple incisions may be made, followed by dilatation, the dilatation being continuous or intermittent according to circumstances. In some canals the wearing of a constant plug will not be well borne; you are obliged, therefore, to dilate from time to time, once or twice a day, or once in two or three days, according to circumstances, but after making free incisions it is important that thorough dilatation should be practiced in order to prevent a re-contraction. This is exemplified in a class of strictures which occur at the orifice of the urethra, for example, from a chancre in that situation. When a close contraction has taken place you have to make free incisions, and it is better to make several incisions in different directions, rather than a single one, and then dilate to a considerable extent; then continue to repeat the dilating process as often as necessary, until all tendency to contraction has been overcome. Half-way measures will not succeed in overcoming these contractions, and the great reason why surgeons have often failed in overcoming cicatricial contractions is that they have not carried the process as far as they ought to do, the incisions have not been sufficiently free, the dilatation has not been carried sufficiently far, or continued sufficiently long.

In the third class of deformities, in which the features are drawn into abnormal positions, the cicatrices may be freely divided, the features replaced in their normal condition, and the chasm thus produced be filled by means of a triangular flap, substantially as in the first class of deformities mentioned, where there is eversion or separation of the orifices of a mucous canal.

In the fourth class, which is characterized by adhesion of apposed cutaneous surfaces, the treatment consists in the free division of the adhesions, throughout their whole extent, and the application of adhesive plasters or bandages, so as to maintain normal separation of the parts, and by their pressure to restrain the exuberant growth of granulations. You have, for example, a case of webbing of the fingers, from the contraction of a burn. After freely dividing the web as far as the normal commissure or a little beyond it, towards the palm of the hand, each finger is to be separately dressed by strips of adhesive plaster, applied with considerable firmness, securing them above the commissure, and making firm pressure with them, so as to restrain the growth of granulations, each side of the wound being separately dressed in that way and the dressing being frequently repeated, you can to a considerable extent overcome the tendency to the reproduction of the web. You will be apt to be somewhat disappointed as to the completeness of the expected result; you will find after making free incisions and dressing the part thoroughly, in spite of your efforts, the web will be to some degree reproduced, and you will be obliged to repeat the incision and repeat that mode of dressing. It may be assisted sometimes by elastic pressure, that is by having a bracelet applied around the wrist, and an India-rubber band to make a continuous pressure, a pressure which will be continuously felt more decidedly than by simple bandages or adhesive plasters.

In the fifth class of cases, where there is fixation of one or more of the extremities in one position, generally attended with marked deformity, and always involving a serious impairment of function, the cicatricial contractions are to be freely divided throughout their whole breadth and thickness, and in many cases by a number of parallel incisions. It is often advantageous, instead of making one incision, and allowing that to gape very widely, to make a number of incisions, so as to cut up the cicatricial surface into small insulated or peninsular masses. When you come to stretch the parts into their normal situation, you will find instead of one very wide chasm, you have a number of small chasms formed, and each portion of the cicatricial tissue, which is left between those incisions, forms a starting point for the growth of new tissue, and it will not have the same tendency to contract as if you divided simply at one part, and and then stretched it with a wide chasm between the incisions. To avoid the danger of gangrene, care is to be taken to avoid disturbing the relations of the insulated cicatricial masses from the subjacent tissues, from which they derive their supply of blood.

In the sixth class of deformities, resulting from cicatricial contraction, the prominent ridges or cords of tissue may be excised or shaved off, and firm pressure made with adhesive plasters and bandages, to prevent their reproduction. During the healing process escharotics may be applied from time to time as the necessity for their use may be indicated.—*Alfred C. Post, M.D., in Annals of Anatomy and Surgery.*

---

HAHNEMANNISM, OTHERWISE CALLED HOMŒOPATHY.—In touching upon the subject of Homœopathy I shall find it hard to be very ill-natured. For it so happened that once, on the occasion of delivering a literary lecture, I found myself unexpectedly assigned to the hospitality of a homœopathic practitioner. If my host had consigned me to a chamber in one corner of a compartment of a hollowed-out mustard-seed; if he had offered me the ten millionth dilution of a drop of coffee on a globule of sugar of milk, and a microscopic fragment of a muscular fibril, with a fraction of a starch cell, and a smell at a pat of oleo-margarine, I might have felt as a patient ought to feel who has been insulted with pharmaceutic infinitesimals equally preposterous and absurd. But I was courteously entreated and handsomely entertained, and in rememberance of that open door and soft bed and well-spread table, I will try to speak of homœopathy, not exactly as Isaac Walton says the angler should treat his frog, "as if he loved him," but at least as kindly as frogs are treated in our physiological laboratory.

The only excuse I can offer for devoting any time to the subject is the fact that it has a certain hold on the community, that it has organizations and institutions which present themselves to the medical student as having a better doctrine and a more effective treatment than what it is pleased to call "the old school," for which "old school" Hahnemann invented the nickname, sometimes used by those who ought to know better, of "allopathy." I require this excuse for introducing the subject, for homœopathy has no *status* among the biological sciences, and has nothing of any practical value, so far as I know, to offer the medical profession. It began by promising to prevent scarlet fever, which it miserably fails to do, and from that day to this it has been a romance of idle promises slipping through the fingers like quicksilver, evaporating without residue like ether from the palm of the hand. If any one of these promises had been fulfilled, if any single remedy brought forward by homœopathy had proved trustworthy and efficacious, it would have been thankfully accepted by the medical profession, which welcomes every method of help unless it shows itself with false pretences, and even then will appropriate any fraction of truth which underlies the deception or delusion.

So far as I can take account of the stock, the present assets of homœopathy consist of a pleasing and sonorous designation, a nomenclature of symptoms, with sets of little phials, containing globules, which are the prettiest and most fascinating of amulets, arranged to correspond with the nomenclature, a collection of "provings" which prove more about the prover than about the questions to be proved, and a doctrine which slips on or off like a kid glove, according to the company in which the practitioner finds himself. Why homœopathy should have so much popular currency in this country as compared with the land of its birth, or with Great Britain, is a curious question. It has been attributed to the state of medical education, but it might be found, I suspect, to be in intimate relations with another very interesting matter, too delicate for me to meddle with here, namely, the potential influence in our community of the imaginative sex, and its psycho-biological leaders and followers.

Hahnemann was not an ignoramus, by any means, but something a great deal worse. He was a hopeless subject of cerebral strabismus, beyond all medical, all surgical, treatment. A squinting eye can be set right, but a squinting brain is too much for the art of gods or men. Whether the strabismus involved the moral as well as the mental faculties of Hahnemann, I will not stop to discuss. But when a man misinterprets all that he reads; when he borrows the most foolish things from the most foolish or erratic writers that he can possibly get hold of, then the less he knows about books the better.

A rogue may have good money in his pocket; but his bills are more likely to be counterfeit than those carried by an honest man.

The Eclectics are the lineal descendants and heirs of the Thomsonians of a past generation, whose botany, as Professor Asa Gray informs me, included not only *lo*belia, but also "*high*belia." The eclectic writers and teachers seem to be a sort of half-armed medical militia, of the class that spells inflammation with one *m*, and whiskey without the *e* in the last syllable. I do not suppose their practice differs very much from that of those whom we call regular physicians. One of their "Professors," who recently left the eclectic for the regular ranks of the profession, gives as his reasons that the original and cardinal doctrines of the eclectic school—opposition to blood-letting and certain mineral remedies on the one hand, and the use of various new remedies on the other—have been largely adopted by the regular school of medicine.

No, gentlemen, there is no exclusive *a priori* method which leads to the successful treatment of disease. You begin in the primrose paths of knowledge which are only preliminary to your real work. Anatomy is no more medicine than a child's dissected alphabet is literature. Physiology and chemistry throw gleams of light here and there on curative methods, but are apt to lead their votaries far away from practice. Pathological anatomy teaches a great deal, but it is, after all, like inspecting what is left of the fireworks on the morning of the fifth of July. It is very pleasant to dissect a muscle, to make a precipitate, to watch a contracting heart, to study a translucent slice of a healthy or diseased organ. These pursuits, sisters of her who presides over health and disease, are the sirens that won over Agassiz and Huxley and Helmholtz to their flowery realms. But just as zoology, chemistry, physiology, histology, are not the science of medicine, so neither is the science of medicine the same thing as the art of healing. To go hastily from the library of old books and the labratory of new experiments to the bedside of disease is imitating the presumption of those rash profligates who, as Thomas Boston says, think they can take a "leap out of Delilah's lap into Abraham's bosom."

But your hardest study must be at the bedside. Your real business will be to save life, to avert disease, to manage it so far as manageable, to save your patients all unnecessary suffering.—*Oliver Wendell Holmes, M. D., in Boston Med. Journal.*

---

Laceration of the Perineum.—In my own opinion, supporting the perineum amounts to but little. The osseous portion of the passage is fixed, the head has to pass, and the perineum must either distend or tear sufficiently to allow it. I prefer to devote my efforts to holding the head well up into the pubic notch, and to pressing the perineum down over it.

When the perineum is rigid, and the head is advancing so rapidly and forcibly as to endanger its continuity, hold back the head to give the perineum time to distend.

I believe also that a large percentage of the lacerations are produced by the shoulder, as we might reasonably expect, when we consider that the curvature of the head makes a beautiful inclined plane, over which the perineum glides without rupture, while the shoulder, being so nearly square, presses almost at a right angle against the perineum, which has contracted close up around the neck after the delivery of the head. At this point of the labor we should recognize the danger that the shoulder may tear right through the perineum, and to prevent it I am in the habit of introducing one or two fingers within the vagina, and, while supporting the head upon the palm, placing the finger tips upon the point of the shoulder. Thus the perineum is pressed from the neck, and slips nicely over the shoulder along the inclined plane made by the fingers.—*G. A. Herman, M.D., in Ohio Med. Journal.*

---

The Cure of Hernia by the Antiseptic Use of Animal Ligature.—The operation may be described in a few words. Antiseptically cut down upon and expose the ring. If the sac is small the peritoneum should not be opened; if large or easily bulging it is much better to exsect it. If this is done, in some instances it will be better to first close the peritoneum independently with a fine continuous suture; otherwise, refresh the pillars of the ring or walls of the opening, and close by sutures. These are covered in by the external tissues which are held in place by superficial stitches; drainage is also used if the abdominal wall is thick.

The dressings are very carefully applied, and union by first intention almost invariably takes place.

At first I used cat-gut and the interrupted suture; now I prefer the parallel fibres of tendons antiseptically prepared, and close the ring very carefully with the continuous suture in close juxtaposition. The two great objections to cat-gut ligatures are, that many times they fail to hold the parts *in situ* sufficiently long, and the knots untie more readily than silk. The parallel fibres of lean animals, like the deer, kangaroo or moose, which latter animal affords tendons nearly two feet in length, quite free from fat, and very strong and firm, do not change as early in the tissues as cat-gut. Therefore I think they are preferable for most surgical purposes.

The continuous suture has a double advantage; it reduces the number of knots to two, which may be a considerable gain, and the collateral swelling of the tissues is equalized by distributing the pressure of the suture throughout its entire length. This insures a uniformity of circulation in the enclosed part, and thereby lessens the devitalization of the tissues, causing a more sure and rapid repair process to take place.

If I am correct in my belief that animal sutures are replaced by bands of living tissue, there is a corresponding advantage in encircling the yielding relaxed parts by closely taken stitches, since thereby we lessen the danger of the return of the hernia by strengthening as well as occluding the ring.

In all the great variety of operations practiced in hernia, one of the prime difficulties and chief causes of failure has been the superabundant peritoneum which easily pouches at its former distended part, is filled with omentum, or intestine, which makes pressure from within outwards upon the closed but weakened ring, and often speedily causes a return of the hernia. Since it now adds so little to the danger of the operation, I think it is generally much better to exsect with care the peritoneum, bring the edges together with a fine over-and-over stitch, so as to leave its inner surface unfolded and smooth. When this has been done it effectually closes the abdominal cavity, and renders the subsequent steps of the operation quite easy. Any hemorrhage which may arise from the wound, or from the refreshing of the walls of the opening, is then external to the peritoneum, and the tendinous structures of the ring are brought into juxtaposition, as stated above.

When drainage is required, I prefer horse-hair. This should be previously prepared in parallel bundles of six, eight or ten hairs, as size may require, held together by carbolized glue or solution of gum, and dried. Then they may be cut in lengths as required, and thus we escape not only the use of knots, which of course are objectionable, but can remove one or more of the hairs as thought wise at each dressing. Owing to the difficulty of securing immobility and close approximation of the dressings it is preferable to secure it with an elastic bandage.

I would claim as advantages for this operation—

1. We are enabled to clearly see each and every step of the operation; blind surgery is bad surgery, as a rule. This want of clear perception has been the source of many mistakes by even experienced operators, and is a great objection to every form of subcutaneous sewing. In my judgment no less can be said of the very ingenious and improved modern process of astringent injection into the ring, as advocated and practiced by Dr. Joseph Warren, of Boston, in his recent work upon hernia. The same objection would hold good of Mr. Spanton's method of approximating the rings by the use of his corkscrew needle, which is left *in situ* for several days.

2. It is the only method with which I am acquainted whereby the hypertrophic elongated peritoneal pouch, which has been a primal factor of failure hitherto, can be removed.

3. Hereby we actually reinforce as well as occlude the ring. In the weakened attenuated condition of the parts in all old cases there is no small gain in securing such effect.

4. Experience has, I think, now demonstrated that this operation may be catalogued among those safely advised, and that femoral and umbilical hernia are no exceptions. I would not exclude children from the class to be benefited; for in them the vital processes are at the best, and when thus cured they are saved from a life-long disability.

It would be difficult to appreciate the amount of suffering produced by hernia; its frequency and its dangers are well known to every medical man. The truss rarely cures the hernia, often even fails to prevent strangulation. The sufferer from hernia is unable to engage in many occupations, and is practically doomed to a life-long disability. Under the guidance of the conservative teachings of our fathers we have relegated to the instrument-maker this whole army of invalids. In the march of progress in surgery, which marks our generation, the cure of hernia may be included among its triumphs, thereby sufferings lessened, life prolonged, and the welfare of the race greatly increased.—*Henry O. Marcy, M.D., Trans. Int. Med. Congress.*

---

Dengue in Columbus, Ga., in 1866.—We attributed the disease to a peculiar poisonous atmosphere, caused by the exposure of the deposits in the bed of the river (which had been covered with water for the last twenty years) to the action of the sun. The water was drawn off in July. The first case of break-bone fever occurred on the 6th day of September.

The disease attacks every one suddenly. The first symptoms are pain and soreness on pressure of the eyes; and pain in the head, sometimes excruciating, with pain in the back, and aching in the back part of the pelvis.

In some cases this is all the pain that is suffered.

In others, in addition to the above, every joint and bone and muscle aches; and when you ask them where it hurts, they will tell you they hurt all over. I heard a little boy who was suffering very much, tell his father both of his thighs were broken. Fever accompanies these symptoms—sometimes of a high grade, but generally the reverse of this. The pulse and skin frequently are very little above the natural standard. During this first paroxysm, which lasts from six or eight to thirty-six or forty-eight hours, according to the treatment used, there is frequently vomiting of the contents of the stomach, followed by vomiting of bile. When the patient goes to bed he wants blankets piled on him, and complains, whenever he moves, that cold streaks run over him. This constitutes the first paroxysm of this painful and disagreeable disease, which, with proper treatment, is almost always the last, though there is occasionally a case where a light attack of fever supervenes on the third or fourth day.

There is an eruption on the skin in almost every case, with troublesome itching.

No matter how mild or severe the attack may have been, the patient is always left in a weak and feeble condition. The mouth tastes horribly. The tongue is generally coated with a cream-like coating, that scarcely ever cleans off as long as the weak, mean feeling stage lasts, which is generally from a week to ten days after they commence walking about. During the greater part of this period there is a perfect loathing of all kinds of food.

In fact, all who had brake-bone fever, say that for a week after they get about, they feel worse than at any time before in the whole course of their lives. Language, they say, fails to describe their feelings.

There is more or less nausea in every case. Persons who are rheumatic, or who have a syphilitic taint, suffer for some time with pain in the various joints. My idea of the disease is, that it is situated in the nervous system, including, of course, its great center—the brain. I do not look upon it as inflammation, but a peculiar morbific effect, the result of a specific poison existing in the atmosphere.

I forgot to mention above, that in the large majority of the cases that have occurred in females who menstruate, this disease has brought on the discharge again, though they may have passed through their regular period ouly a week or ten days previous. I attended one lady who had an infant only five weeks old. The discharge came on; and went regularly through, as it did in others. Dr. Terry told me that he had two patients, one had seen nothing for three and the other five months. During an attack of brake-bone fever, both had a return of their period.

The treatment of this disease is very simple. When called to a case, if I find that the patient has not taken something to act on the bowels, I generally give a mercurial purge if the pains are not very severe; but if they are, I give a full dose of epsom salts in pepper tea. I use the pepper tea on account of chilliness complained of, though sometimes, when the stomach is very irritable, I give simply a seidlitz powder every hour until the bowels are moved. My object is to have the bowels acted on as soon as possible, so that the system will be prepared for the introduction of opium, which is the great remedy in this disease. As soon as two or three actions have been produced, I give a pill composed of opium and camphor, one grain each. This is repeated in two hours, and then again every four hours until four or six pills are taken.

If, on the second or third day, fever returns, a seidlitz powder is ordered every two hours until an action is secured. This, however, seldom occurs when opium has been used. Quinine is hurtful in this disease; it increases the nausea and general distress.

Large mustard plasters used at any time, but particularly in the commencement of the attack, afford great relief and comfort; also hot foot baths and hot applications to the feet. Let the patient drink at all times as much hot or cold lemonade (*without ice*) as he desires.

After this I tell the patient, if he feels pain anywhere, to take two spoonfuls of paregoric every hour until relieved; also to eat and drink whatever he wishes, and to go where he desires, feeling sure from personal experience that they have no disposition to commit excesses in either.

I have not yet seen or heard of a relapse from this disease, nor a return of it in the same person during the same season.

I have not lost a case of it, nor have I heard of a death from this cause, unless some old persons who died here last fall had it.

The disease, in my opinion, is not contagious, but is caused by a peculiar poison in the atmosphere.—*Jno. E. Bacon, M.D., in Proceedings Atlanta Medical Society*, 1867.

---

LOCAL ANÆSTHESIA IN NEURALGIA.—We are not, as a rule, addicted to taking much stock in cures, in fact, we generally pass over with a feeling allied to contempt, that department of medical literature containing prescriptions for various ailments; whether they emanate from the pompous gold-spectacled professor, or the eight-by-ten cross-roads doser; and we are prone to accept nothing without a "reason given for the hope that is within them." But for once we will deviate from our established rule, for once we will make a plain statement of facts without offering any theory for their elucidation, leaving to our readers the pleasure of drawing any deductions that may suit their fancy.

In the spring of 1869 we, our "ain sel," had the most severe attack of facial neuralgia which it has been our lot to witness in more than eighteen years of practice; for two weeks we had to confine ourselves to a darkened chamber, and the lighest foot-fall on the floor caused us the most excruciating agony. All the remedies, local, general, regular and irregular, were tried without any abatement of the trouble. One side of our face was terribly swollen, so much so that it was impossible to extract a decayed molar, to which we charged all our suffering, and it seemed as if we were destined to shuffle off this mortal coil by exhaustion from pain and want of sleep. We finally concluded to incise the swollen jaw, thinking there was probably an abscess about the root of the decayed tooth, and as the parts were so extremely sensitive, and moreover, having a vague dread of chloroform, we thought we would try local anæsthesia, by evaporating ether on the surface until the part was frozen. Our attendant complied with our instruction, and the spray was turned on. The first sensation was one of cutting pain, gradually subsiding until when congelation took place we felt perfectly easy, and ordered the cutting operation deferred. Then for fifteen hours we slept the sleep of the righteous, and when we awoke found the *rubor et tumore calore cum dolore* entirely vanished, and we arose and went about our business, and to this good day, although we carry a perfect cabinet of carious teeth in our mouth, have never had a neuralgic twinge or a touch of that "hell of a disease," a toothache. Well, to be honest about it, we did not at the time give the process any credit for the cure, we thought the attack had about spent its force and was going to get well anyway, and we paid but little attention to the matter for a year or more, when a relative, who was visiting us, took a spell of neuralgia, with which he had for over a year been periodically afflicted, rarely passing a month without an attack. To give him present ease, for we did not think of any permanent benefit, we tried the spray all along the track of the affected nerve until it turned the skin white; the relief was immediate, and he has since informed me permanent.

Since then we have used it in fifteen or twenty cases with uniform success, never having to make more than two applications, and it came to be a stock remedy, and we thought that in all probability, was so with most physicians, for we remember that when Richardson first introduced it, (like all new things in medicine, it was vaunted for everything,) and would probably have still thought so, if a gentleman had not called on us sometime ago to know if we hadn't a new treatment for neuralgia, and stated a couple of years ago he was on a steamboat and was suffering with that disease, when one of our patients informed him that he was cured by some sort of a freezing process, and advised him to try it. When the boat reached Louisville, he called on two or three dentists, and three of the most distinguished surgeons of the city, and they told him they knew of no such remedy for neuralgia, and advised him not to have anything of the kind done. On hearing his story, we looked over our old volumes of medical journals and found not a single allusion to local anæsthesia as a remedy for neuralgia.

Now we must confess that all this sounds very much like the story of the superanuated clergyman who accidentally, while in the West Indies, discovered a cure for consumption, etc., only we don't want any one to send a stamp for particulars. Any physician can purchase a hand-ball atomizer for one and a half dollars and try it themselves. They may use either rhigolene or ether, and it will only be necessary to let the spray play upon the part until the skin turns white. We promised to offer no theory for its action, but we will venture this opinion: that the intense cold by its revulsive effect causes a complete change in the nutrition of the nerve, what this change is, we will not at present venture to assert, only hoping that others who have better opportunities will give the matter a trial and fully test its merits.—*J. T. McColgan, M. D., in Southern Practitioner.*

---

Pompeian Surgery.—An interesting sketch of the surgical instruments collected at Pompeii, and preserved in the museum at Naples, has been given in a recent number of the *Revue Medicale* by M. Jouin. At the museum they are arbitrarily divided into surgical and obstetrical instruments, but there is little in the latter to suggest that they were intended for obstetrical purposes. A pair of forceps, for instance, classed among the obstetrical instruments, does not appear to have been ever intended for such use. The blades are twenty-one centimeters long, they cross one another, and are articulated by a pivot; the handles are curved; they are apparently similar to the instruments now used to remove sequestra, etc. There is, however, a tube clearly intended for injections into the vagina. It is twelve centimeters long; one extremity is manifestly designed to receive the nozzle of a syringe, while the other is perforated with holes, one terminal and the others arranged in two circles, so that the jet may be broken and spread, just as in the similar tubes in use at the present day.

There is also a very ingenious trivalve speculum, evidently intended for the vagina, so made that the three blades can be opened or closed simultaneously. There is a rectal speculum, fifteen centimeters long, composed of two blades which can be closed or opened by means of a pivot placed in the centre of the instrument, and presenting the type according to which all similar specula are made at the present day.

There are catheters for women, straight, made of silver. A curious instrument, which consists of an iron rod, at the extremity of which is a small rectangular plate of iron, two centimeters long and three wide, fixed to the rod at an angle of 135 degrees, is exhibited as a cautery for wounds, the Italian surgeons believing that it is intended to cauterize deep structures, such as the uterus or pharynx. The perfect resemblance in form to the laryngeal mirrors now in use suggested to M. Jouin that it may really have been intended for a similar use, to examine deep structures, if not the larynx. Catheters for men have also been found; they are twenty seven centimeteres long, and have a very peculiar double curve like a very long S. M. Jouin thinks that this form shows a very imperfect knowledge of the real curves of the urethra; but under ordinary circumstances this is nearly the form of the urethral canal, although the introduction of such instrument may have been a matter of some difficulty, its shape would facilitate the emptying of the bladder.

Among the other instruments are a metalic trocar in two pieces, similar to those in use at the present day, bistouries, very large lancets, various forms of stylets, curved and straight, some probably intended for the examination of carious teeth, curette spatulas, small forceps, and various needles and hooks. There are also some surgical cases with instruments and cases for pills, ointments, etc. All these instruments were found in one house, and in number they will certainly bear comparison with those possessed by an average practitioner in a provincial town at the present day.—*Lancet—Med. Gazette.*

ACUTE TONSILLITIS.—I shall now bring before you an example of a slight affection, but one which sometimes gives considerable trouble. It is a case of tonsillitis, or, as it is commonly called, quinsy. Inflammation of the tonsil is generally the result of a partial exposure, especially of the neck, to cold. This patient, who, as you see, is an anæmic, unhealthy-looking girl, contracted the disease while in the house under treatment for amenorrhœa. Tonsillitis may be the consequence of some general disease, as, for instance, scarlet fever, but, as I have said, it is generally the result of some local cause.

In this case the disease has passed its height, but there is still considerable enlargement of the tonsils, especially on the right side. When the patient was first taken sick, the amount of swelling was so great, and she had, in consequence, so much difficulty in opening her mouth, that it was almost impossible to get a good view of the fauces. You will notice that the tonsils are simply swollen and reddened; there is no deposit upon them. It is very important to examine carefully the throat for the presence of false membrane, the inflammation is probably of a simple character. Do not mistake, as is sometimes done, the secretion of the tonsillar glands for membrane. You can readily understand that when the tonsils are inflamed there may be, in earlier stages especially, an increased secretion, and you will often find a cheesy, curdy mass on the tonsils; but this does not indicate the presence of diphtheria.

The enlargement of the tonsils may become so great as to cause marked interference with deglutition, and also give rise to partial deafness from interference with the Eustachian tube. Even liquid food is often swallowed with great difficulty. The swelling also, by closing up the passage to the larynx, renders the respirations exceedingly labored and difficult, and may give rise to cyanosis from deficient aeration of the blood.

As a general rule, tonsillitis runs a favorable course. It reaches its greatest height in two or three days, and then there is a rapid subsidence. In other cases, however, the course is less favorable. We have the enlargement and then the breaking down of the tonsil, forming an abscess. It is sometimes necessary to puncture these abscesses. In doing so, some little care should be taken, as several large vessels run over the tonsil. It is better to make the puncture towards the posterior part, as the vessels are on the anterior portion. In some cases the gland ruptures spontaneously. In other cases we have left a chronic condition, in which the gland remains permanently enlarged. It is often necessary in such cases to resort to surgical means to get rid of it, either by caustics or cutting off the tonsil with an instrument made for this purpose. Many of these cases of chronic enlargement will, I think, yield to nitrate of silver in solution, or to the tincture of iodine.

In this case the treatment was very simple. There was no necessity for active treatment. Formerly it was the habit in cases of tonsillitis to wash out the throat with a strong solution of nitrate of silver. I believe that if this is done in the commencement it may abort the the attack; but if not done at the very beginning it will do harm. If the symptoms are not very active, it is better not to have recourse to this method, but to use milder remedies.

As this patient needed a purgative, she was given a dose of blue mass, followed by a saline. She was then placed upon the tincture of chloride of iron, which acts both locally and constitutionally. Great benefits will sometimes be obtained from gargles. The parts will be kept clean and free from the viscid, tough mucus which frequently causes distressing symptoms. Chlorate of potassa with myrrh and muriatic acid will be found an efficient gargle after the acute period of the disease has passed. If the disease becomes chronic, and the glands show no disposition to undergo diminution, tincture of iron, tincture of iodine, or solutions of nitrate of silver may be applied. After this disease has occurred once, it it is apt to leave behind it a tendency to repetition. You will often meet patients who have an attack of tonsillitis every year, or after every exposure to cold.—*James H. Hutchinson, M.D., in Philadelphia Med. Times.*

A Case of Lodgment of a Foreign Body in the Cavities of the Nose, Orbit, and Cranium, where it Remained Five Months; Removal by Operation; Subsequent Trephining for Pus in the Brain; Death.

The above is the title of a remarkable case reported by Henry D. Noyes, M D.,

A farmer aged nineteen years, in robust health was wounded in the face by the explosion of a gun. He was knocked senseless, and something was heard to whiz past his companion as the gun exploded. The wounded boy remained unconscious about four days. When he came under the care of Dr. Noyes, five months later, for the performance of a plastic operation, he was apparently in good health, with a deep scar extending from the middle of the fractured and depressed nose along the inner canthus of the right eye upwards and outwards to about the middle of the brow. The upper lid was closed, the palpebral fissure was below the normal level, a fistulous opening existed at the inner border of the eyebrow, the right eyeball was atrophied and immovably adherent to the tissues at the inner portion of the orbit, and from the nostrils there was an offensive discharge, which suggested the presence of carious bone. The nasal bones were badly sunken, and the tip of the nose turned up. On exploring the nasal cavity, by the aid of a mirror and gaslight, a foreign body was discovered in the middle of the right side, which clicked like iron when touched with a probe. The finger passed through the mouth into the posterior naris, came in contact with this body, and the patient submitted to a pretty vigorous attempt at its extraction, when it was seized in front by a pair of strong polypus forceps, and pressed upon from behind with the forefinger of the other hand carried up behind the soft palate. The mass could not be stirred, and he was soon convinced that it extended into the orbital cavity.

A week later it was removed by an extensive operation. It proved to be the breech-pinof the gun and measured $4\frac{7}{16}$ inches in length; its breadth was about a half inch and its weight was two ounces, five drachms and twenty-five grains, or about eighty-four grammes. The patient did fairly well after the operation, but soon began to show symptoms of suppurative inflammation in the brain; the skull was therefore trephined, and a brain abscess opened. The patient died thirty-nine days after the removal of the foreign body.—*Am. Journal of Medical Sciences.*

---

Urethral Caruncle and Cystitis.—This patient then has a urethral caruncle. It consists of hyperplastic papillæ of mucous membrane lining the urethra. We may have a large number of these papillæ enlarged. These growths occur more frequently in stout women. Want of cleanliness, vaginal leucorrhœa, or urethritis may cause this condition. The treatment consists in removing the growth. Put the patient under ether, separate the labia, take either an ordinary dressing forceps or one of the various forms of urethral specula and expose the growth. Then take a double tenaculum and hook in the growth, lift it up so as to get at the base and apply a small looped wire. By twisting this tightly the growth may be removed, or it may be burned off by the Pacquelin cautery at a dull heat. I usually snip off the growth with scissors and cauterize the base with Pacquelin or fuming nitric acid to prevent hemorrhage and its recurrence. The after treatment consists simply in moderate irrigation. If there is much pain apply ice-water compresses over the vulva. It is well to perform dilatation of the urethra before cauterizing, as vesical tenesmus is liable to ensue. Be careful, however, not to overdilate the female urethra. The little finger is always sufficient, and permanent incontinence will not then follow this moderate distention.

This patient is also troubled with chronic cystitis. The condition is very disagreeable to treat and very difficult to cure. It may come from a cystocele, retention of urine, from exposure to cold or as the result of the parturient act from pressure, and from anterior displacement of the uterus on the bladder. The symptoms of cystitis in the female are frequent desire to pass water, scalding in act of micturition and sediment in the urine, which is generally turbid and ropy.

The most marked symptom is the frequent desire to pass water and the scalding. Patients have told me that they will get up twenty times in the night and pass only a few drops at each time. These patients soon become anæmic. The frequent micturition is relieved by moderately dilating the urethra as already described. You can cure the cystitis by internal remedies such as balsam of copaiba, fluid extract or infusion of pareira brava, uva ursi, and by one of the best triticum repens, with benzoate of soda. Such cases are always tedious in proportion to the length of time the disease has existed.—*Paul F. Munde, M.D., in Med. Gazette.*

---

AUGUSTA HEALTH BOARD.—"The Fourth Annual Report of the Board of Health of the City of Augusta, Ga.," is a pamphlet of some interest. Here is a well-located town of twenty-five thousand inhabitants, not overcrowded, with no tenement house population—and yet with an annual mortality rate of nearly three per cent.; the rate for whites being 22.82 and for the colored 38.85 per 1,000. Of this heavy death-rate over one-third falls on the children under five years of age. Clearly, Augusta must be a bad place for raising a family. Among adults the great mortality is due to consumption, pneumonia and typhoid fever. These people are being poisoned in the usual and regular way. The very encouraging and satisfactory feature of the case is that the health authorities do not in the least attempt to deny or to conceal the facts—they recognize them clearly and state the remedy in unmistakeable terms. This remedy is a proper system of sewerage and drainage. A still better sign is the fact that upon the question of adoption or rejection of the proposition to expend four hundred thousand dollars for a system of sewerage and drainage, and an increase of the water supply the people of Augusta decided in the affirmative by more than a two-thirds majority. A systematic house-to-house inspection of the city has been carried on throughout of the year, resulting in the report of a large number of nuisances and dangers to the public health. In short, the Board of Health seems to be active and intelligent, and its work deserves the commendation and support of all intelligent citizens. —*Sanitary Engineer.*

Unfortunately the benefits anticipated from the improved system of sewers and the new water supply are not to be realized—at least, for the present—as a decision of the courts prohibited the issuance of the bonds, by the city, necessary to execute the proposed plans.

---

THE THERMOMETRIC BUREAU.—The number of thermometers examined is more than double the number examined during the preceding year.

Compared with last year's work the figures are as follows:

| | 1880-81 | 1881-82 |
|---|---|---|
| Physicians' thermometers examined | 1667 | 3811 |
| Other thermometers examined | 290 | 741 |
| Total | 1957 | 4552 |

In the year ending May 31st, 1882, we received twenty thermometers which were broken in transit to us, and four thermometers were broken in our hands. In every case of the the thermometers broken on arrival, it appeared as though proper packing would have ensured safe transmission. No thermometers were broken when entrusted to the hands of our New York messenger, and nine-tenths of the other breakages occurred in the mails. The four broken at the observatory, one-tenth of one per cent. of those examined, represent the risk to which a large number of thermometers are exposed in our manipulations, and it is not probable this percentage can be lessened.

The improvement in the manufacture of clinical thermometers in this country continues, and the thermometers we receive which are most misleading in their indications are those which come in from private practice, and which have been in use for a year or more.

We have been much encouraged in this department of the observatory work by the cordial endorsement given to it by the medical press.—*Second An. Report of Thermometric Bureau, Yale College.*

---

TREATMENT OF HEMORRHAGE FROM THE PALM.—1. Hemorrhage from the palm should be treated by ligating or twisting the bleeding vessels in situ, when this can be done without undue disturbance of the tendons and other tissues.

2. This being impossible, compression should be applied in the wound by means of a graduated compress or conical cork, flexion and pronation of the arm being also employed, or the hand may be bound tightly over a ball of wood or cork.

3. Acupressure may be used to the radial artery, but it is not safe to compress the ulnar in this way, as the nerve is in close proximity on its inner side.

4. Other measures failing, ligation of the main vessels should be performed.

5. Owing to the frequeut anomalies of the brachial artery, it is better to ligate the radial and ulnar immediately above the wrist, than to tie the brachial itself.

6. The hand should be wrapped in cotton wool fixed upon a splint and placed in a sling.—*Randolph Winslow, M.D., in Maryland Med. Journal.*

---

SCROFULOUS GLANDS.—1. That scrofulous neck and glandular scrofula elsewhere owes its origin to local causes, with or without hereditary tendency.

2. That by efficient sanitation such maladies may be prevented, even in the most susceptible.

3. That scrofula, when established, even in severe forms, lends itself easily to radical methods of cure.

4. That, especially by surgical dissection, the disease may be promptly removed, and the patient cured in a few weeks.

5. That by this method disfigurement, if not wholly averted, is reduced to the least amount.

6. That the subsequent health is after such procedure re-established in a way that is not possible under any other mode of cure, and that the risk of future phthisis or any other malady caused by septic absorption is averted. —*Clifford Allbutt, M.D.—Int. Med. Congress—in Am. Practitioner.*

---

Analysis of Milk.—The simplest and most rapid process for the analysis of milk is that proposed by M. Adam some years since to the French Academy of Sciences. The apparatus consists essentially of a glass tube, holding forty centimetres, having a stopper above and a glass tube and stop cock below. Into this is introduced, first, ten cubic centimetres of alcohol at 75° C., containing 1-200 of its volume of caustic soda; secondly, ten cubic centimetres of neutral milk; and, thirdly, twelve cubic centimetres of pure ether. The tube is closed, well shaken, and allowed to rest for ten minutes. Almost instantly two layers are formed, sharply separated—an upper, limpid layer, containing all the butter; secondly, a lower opalescent layer, containing all the lactose and all the casein. The lower layer is then drawn out, with the exception of about one centimetre. That which remains is then shaken up with the upper layer. This buttery solution is allowed to run out into a porcelain capsule, and is washed with ether to collect the fat, evaporated and weighed. The difference gives the weight of the butter increased by one centigramme in consequence of adherent lacto-caseous matter. If the ether is then evaporated in another capsule, the direct weight of the butter is ascertained. In order to separate and estimate the lactose and casein, the liquid first drawn off is diluted with distilled water to 100 cubic centimetres, and ten drops of acetic acid are added. The casein then separates in a flocculent precipitate like that of nitrate of silver. After allowing it to stand for five minutes, it is poured upon a dry filter, which is kept covered to prevent evaporation. Thus 94 to 96 per cent. is collected of a liquid which contains only the salts of the milk, acetate of soda formed during the process, and lactose. The latter is then estimated by Fehling's solution. If a known volume is then evaporated to dryness, the amount of lactose can also be distinguished by two weighings, one before, the other after, incineration, deducting from the weight thus obtained that of the acetic acid combined with the soda. This casein is washed two or three times with distilled water and the filtering paper containing it is strongly pressed between two layers of paper to flatten it as much as possible, and it can then be dried in a few minutes. The weight of the filter before and after the operation gives that of the casein. Or the casein may be detached from the filter, dried and weighed. These operations are easily executed in an hour and a half, and if at the beginning an additional ten cubic centimetres of milk is placed to evaporate with two drops of acetic acid, the amount of dry residue of water and of ashes may also be ascertained. Five cubic centimetres of milk will suffice for the analysis, and the apparatus is very portable. The New Jersey State Board of Health, however, gives preference to the analysis of Rittenhausen. *John Morris, M.D , in Maryland Med. Journal.*

---

ANOTHER DROP IN THE BUCKET.—The following monument of epistolary art, and record of the aspiring plans of a would-be medical teacher was recently received by the publishers of the *Medical Gazette :*

COLLEGE OF PHYSICIANS AND SURGEONS,
JOPLIN, MO., 5, 28, 1882.

GENTS.—Please send *price list of Doctors,* and *Drugist. Names.* by states as I want to mail *several thousand* Annual *Catalogues* to the *Profession* all over the U. S. A. and canady. I am starting an embriotic Pioneer-Medical College and I must, of necessity, noise it around to the world to make it pay me. An early answer will greatly oblige your Respectfuly, &c. J. C. PETIT, M.D., Dean.

---

ABSORPTION OF SEQUESTRA.—M. Vrignal has lately made a series of experiments on the absorption of sequestra. He has determined that a sequestrum covered with pus will not be absorbed, whilst one enveloped in granulations will be. A fragment of bone (a bone peg) being driven into the tibia of a rabbit was almost entirely absorbed.—*Gazette des Hopitaux.—St. Louis Med. and Surg. Journal.*

